# Science-based approach for credible accounting of mitigation in managed forests

**DOI:** 10.1186/s13021-018-0096-2

**Published:** 2018-05-17

**Authors:** Giacomo Grassi, Roberto Pilli, Jo House, Sandro Federici, Werner A. Kurz

**Affiliations:** 1 Giacomo Grassi, European Commission, Joint Research Centre, 21027 Ispra, VA Italy; 2 Roberto Pilli, European Commission, Joint Research Centre, 21027 Ispra, VA Italy; 3 Department of Geographical Sciences, Cabot Institute, University of Bristol, Bristol, BS8 1SS UK; 4 Food and Agriculture Organization (FAO) Consultant, 00153 Rome, Italy; 5 Natural Resources Canada, Canadian Forest Service, Victoria, BC V8Z 1M5 Canada

**Keywords:** Forest, Reference level, Sink, Bioenergy, European countries, Carbon Budget Model

## Abstract

**Background:**

The credibility and effectiveness of country climate targets under the Paris Agreement requires that, in all greenhouse gas (GHG) sectors, the accounted mitigation outcomes reflect genuine deviations from the type and magnitude of activities generating emissions in the base year or baseline. This is challenging for the forestry sector, as the future net emissions can change irrespective of actual management activities, because of age-related stand dynamics resulting from past management and natural disturbances. The solution implemented under the Kyoto Protocol (2013–2020) was accounting mitigation as deviation from a projected (forward-looking) “forest reference level”, which considered the age-related dynamics but also allowed including the assumed future implementation of approved policies. This caused controversies, as unverifiable counterfactual scenarios with inflated future harvest could lead to credits where no change in management has actually occurred, or conversely, failing to reflect in the accounts a policy-driven increase in net emissions. Instead, here we describe an approach to set reference levels based on the projected continuation of documented historical forest management practice, i.e. reflecting age-related dynamics but not the future impact of policies. We illustrate a possible method to implement this approach at the level of the European Union (EU) using the Carbon Budget Model.

**Results:**

Using EU country data, we show that forest sinks between 2013 and 2016 were greater than that assumed in the 2013–2020 EU reference level under the Kyoto Protocol, which would lead to credits of 110–120 Mt CO_2_/year (capped at 70–80 Mt CO_2_/year, equivalent to 1.3% of 1990 EU total emissions). By modelling the continuation of management practice documented historically (2000–2009), we show that these credits are mostly due to the inclusion in the reference levels of policy-assumed harvest increases that never materialized. With our proposed approach, harvest is expected to increase (12% in 2030 at EU-level, relative to 2000–2009), but more slowly than in current forest reference levels, and only because of age-related dynamics, i.e. increased growing stocks in maturing forests.

**Conclusions:**

Our science-based approach, compatible with the EU post-2020 climate legislation, helps to ensure that only genuine deviations from the continuation of historically documented forest management practices are accounted toward climate targets, therefore enhancing the consistency and comparability across GHG sectors. It provides flexibility for countries to increase harvest in future reference levels when justified by age-related dynamics. It offers a policy-neutral solution to the polarized debate on forest accounting (especially on bioenergy) and supports the credibility of forest sector mitigation under the Paris Agreement.

## Background

### Forest mitigation in the context of the Paris Agreement

In order to achieve the Paris Agreement’s long-term goal of keeping “the increase in the global average temperature to well below 2 °C” [1], countries “should take action to conserve and enhance, as appropriate, sinks and reservoirs of greenhouse gases […], including forests” (Art 5.1 of the Paris Agreement) and “are encouraged to take action to implement and support […] activities relating to reducing emissions from deforestation and forest degradation” (Art 5.2).

A high expectation for forest mitigation emerges both in countries’ climate targets (i.e., the nationally determined contributions, NDCs), where forests are assumed to provide up to a quarter of planned emission reductions by 2030 [2], and in estimates of land-based mitigation potential [3] and pathways to achieve 2° [4]. Globally, most of the cost-effective mitigation potential is expected from avoided deforestation in the tropics [3]. However, the management of temperate and boreal forests also offers a rich portfolio of effective mitigation options (e.g. [5]), including conserving and enhancing the existing sink and using wood-based products to reduce emissions in other sectors through material and energy substitution [6].

Furthermore, when countries “account” for the impact of mitigation actions towards their NDCs (including the forest sector), they “shall promote environmental integrity, transparency, accuracy, completeness, comparability and consistency, and ensure the avoidance of double counting” (Art 4.13 of the Paris Agreement).

### The challenge of credible accounting the sink in managed forests

In order to achieve the most cost-effective mitigation and to ensure no displacement of emissions among GHG sectors, countries are required (or encouraged, for developing countries) to commit to economy-wide mitigation targets (Art 4.4 of the Paris Agreement). In these kinds of targets, the fungibility across sectors requires that mitigation contributions from different GHG sectors are consistent and comparable, i.e. “one ton of carbon” in one sector should correspond to “one ton of carbon” in other sectors. In principle, within an economy-wide target expressed relative to a base year (or baseline), future net GHG emissions from all sectors should be compared to the net GHG emissions of the base year (or baseline), and any resulting reduction of emissions may be considered to reflect changes in management (i.e., in the type and magnitude of activities, due to policy or market drivers) and consequently a mitigation effort. However, this approach does not necessarily work for existing forests.

Assessing the mitigation outcomes in the forest sector is more complex than in other GHG sectors (e.g., energy, agriculture). This is because it can be hard to disentangle the simultaneous natural and anthropogenic processes that determine forest-related fluxes. Moreover, unlike other sectors, future emissions and removals in forests can change over time as a result of forest characteristics such as age-class distributions, which are largely determined by past forest management and natural disturbances [7].

Under the United Nations Framework Convention on Climate Change (UNFCCC), this complexity has been addressed through a distinction between “reporting” and “accounting” of GHG fluxes, which is unique for the sector “land use, land-use change and forestry” (LULUCF, [8, 9]).

“Reporting” refers to the inclusion of estimates of anthropogenic GHG fluxes in national GHG inventories, following the methodological guidance provided by the intergovernmental panel on climate change (IPCC). As a pragmatic solution for reporting anthropogenic fluxes under UNFCCC, the IPCC developed the “managed land proxy”. This assumes that all GHG fluxes occurring on land identified by the country as “managed land” are “anthropogenic” [10, 11]. The GHG inventories reported under the UNFCCC should, in principle, aim to reflect “what the atmosphere sees” in managed lands, within the limits given by the method used and the data available.

In the context of mitigation targets (e.g. under the Kyoto Protocol and the Paris Agreement), “accounting” refers to the comparison of emissions and removals with the target and quantifies progress toward the target. Targets are typically expressed relative to the emissions in a base year (or baseline), thus the accounted mitigation outcomes should reflect genuine deviations from the activities generating emissions in the base year (or baseline). For the LULUCF sector, specific “accounting rules” may be applied to filter reported flux estimates with the aim to better quantify the results of mitigation actions (and implicitly to reflect a deviation from a historical or business-as-usual management). LULUCF accounting then produces “debits” or “credits” (i.e. extra emissions or extra emission reductions, respectively) that count toward the target. This should provide appropriate incentives/disincentives for beneficial/detrimental actions and help assessing the effectiveness of policy measures [12]. At the same time, credibility in LULUCF accounting is required to give confidence that credits are not earned when mitigation has not occurred.

Note that the “filtering” done by LULUCF accounting may be important in the context of the NDCs—to help ensuring comparability and consistency across sectors and countries—but it does not necessarily apply for assessing the “balance” between global anthropogenic GHG emissions and removals in the second half of this century (Art. 4.1 of the Paris Agreement). The “balance” refers more to “what the atmosphere sees”, reflecting the collective countries’ progress, rather than to the impact of individual country’s mitigation actions. Although the modalities for assessing “balance” under the Global Stocktake (Art 14) are still under discussion, all the “anthropogenic” removals reported for managed lands in GHG inventories are expected to be taken into account [13], including those that do not necessarily reflect a deviation from historical management.

For land that experiences human-induced forest conversions (i.e. afforestation, reforestation or deforestation), the quantification of the mitigation actions is straightforward because the GHG fluxes are clearly result of direct human actions. Thus under the Kyoto Protocol all forest conversion fluxes reported under UNFCCC are accounted towards mitigation targets.

However, the problem of disentangling the impact of mitigation efforts in extant forests (i.e. “forest remaining forest” in country GHG inventories, including areas classified as forest for at least 20 years) is more complex. Legacy effects, resulting from past natural disturbances and forest management activities, determine today’s forest age-class distribution and in turn future emissions and removals [7]. In this situation, countries could be “penalized” if forests are getting older, because the net sink may decrease due to age-related effects (e.g., lower increment generally associated with older forests) and not with changes in management. Conversely, countries may benefit from increasing sinks in young existing forests without implementation of deliberate changes in forest management that happen subsequent to the base year (e.g., sinks could be due to recovery from past disturbance).

Despite several efforts to develop widely acceptable accounting rules, assessing the mitigation outcomes in extant forests has always been a controversial topic during climate negotiations, affecting adversely the credibility of the forest sink mitigation and its comparability with other GHG sectors [13–16]. The solution adopted under the 1st commitment period of the Kyoto Protocol (2008–2012)—i.e. a simple cap applied to the GHG flux of extant forests, to reflect that this flux was not entirely anthropogenic—has been widely criticized for limiting the incentive for further mitigation action [15].

To better reflect the deviation from a business-as-usual management of mitigation actions, the concept of a projected (forward-looking) “reference level” was developed. The reference level provides a counterfactual business-as-usual scenario of what future net emissions would be, against which the actual future net emissions can be compared [7, 12, 17]. If mitigation actions beyond the business-as-usual management resulted in changes in net emissions, then this will be reflected in the difference between the business-as-usual reference level and the actual emissions.

This concept was adopted for accounting the mitigation by extant forest under the 2nd commitment period of the Kyoto Protocol (KP-CP2, 2013–2020) [18], with an additional “cap” on any resulting accounted “credit” which is equal to 3.5% of total emissions (in all sectors) in the base year (e.g. 1990). To this aim, Annex 1 (i.e., developed) countries submitted projected forest reference levels in 2011 following a specific UNFCCC guidance [19]. Importantly, these reference levels under the Kyoto Protocol included the projected impact of not only age-related dynamics, but in some cases also of the assumed future implementation of domestic policies adopted by 2009. For example, pre-2009 policies allowing increasing harvest up to a certain % of the increment, or planning new biomass power plants (which require additional harvest), were included in some reference levels [20]. This was controversial, as it opens up the possibility of inflating future expectations of emissions, in order to make targets easier to meet [15, 16, 21–23]. Despite these concerns, reference levels have been generally seen as a step forward in accounting for mitigation through the forest sector.

### Controversies regarding future forest accounting rules: the EU case

In the EU, forests have recently been accumulating more timber volume (growing stock) than was harvested [24]: for the period 2000–2016, they acted as a average net sink of ≈ 430 Mt CO_2_/year, equivalent to about 9% of total EU GHG emissions over the same period [25]. Most of this sink (≈ 380 Mt CO_2_/year) occurs in the “forest remaining forest” category, with the remainder in the “land converted to forest” (including afforestation or reforestation) category. Since forests are getting older in most EU countries, and because older forests grow more slowly, the extent to which this sink may be sustained in the near future is uncertain [26]. In addition, new policies will likely increase harvest (e.g., [27]), leading to a possible reduction of the sink over the next few decades.

In the context of the discussion on the inclusion of the LULUCF sector in the EU 2030 climate targets [28] and thus in the EU NDC to the Paris Agreement, the approach and criteria to set the projected “forest reference levels” (FRLs) for post-2020 have triggered controversy and much debate, especially in relation to forest bioenergy (e.g. [15, 29]). The controversy is, in simple terms: if the forest sink decreases as a result of an increase in harvest driven by policies (e.g. support for biomass use for energy, leading to increase in wood demand), should this reduced forest sink be reflected in the accounting toward the EU NDC target?

Some country and forest stakeholders consider that any increase in harvest in the context of existing “sustainable forest management policies” (e.g., harvesting potentially up to the full forest growth increment) should be allowed without generating accounting “debits” (see e.g. [29]). The proponents of this approach argue that they have growing forests due to their past management and thus should be able to harvest this growth so long as they are not reducing stocks. Enabling such an increase in harvest without debits would be similar to the approach implemented in the KP-CP2, i.e. allowing for inclusion in the reference level of a projected (assumed) policy-driven increase in harvesting (i.e. a “demand-side” projection), and the related reduction in the net carbon sink. However, does this approach truly reflect a genuine deviation from a business-as-usual management? And is this approach comparable with the way GHG emissions are treated in other sectors?

### Aim of this study

The aim of our study is to present a credible approach for the accounting of forest mitigation that is consistent and comparable to the way GHG emissions are treated in other sectors, while avoiding potentially “unfair” outcomes associated with the possible future decline of the forest sink (or the increase of forest harvest) because of age-related dynamics.

We first assess the EU-level impact of including the assumed future effect of policies in the forest reference levels under the Kyoto Protocol, based on the country GHG reporting available so far (2013–2016). Based on the lessons learnt under the Kyoto Protocol, and building on a previous methodological report [30], we propose our approach for a more credible accounting of the forest sink mitigation outcomes and illustrate a possible method to implement it. We then apply this method in the EU, using the Carbon Budget Model [31, 32], and discuss the implications of our findings in the context of EU policy, the Paris Agreement and the recent debates on bioenergy accounting.

## Results

### Analysis of reference levels under the Kyoto Protocol (2013–2016)

The calculation the forest reference levels under the Kyoto Protocol CP2 (called “forest management reference levels”, FMRLs), submitted and technically assessed in 2011 [20], considered the effects of age-related stand dynamics and implicitly allowed for inclusion of the assumed future implementation of domestic policies that had already been approved.

**Fig. 1:**
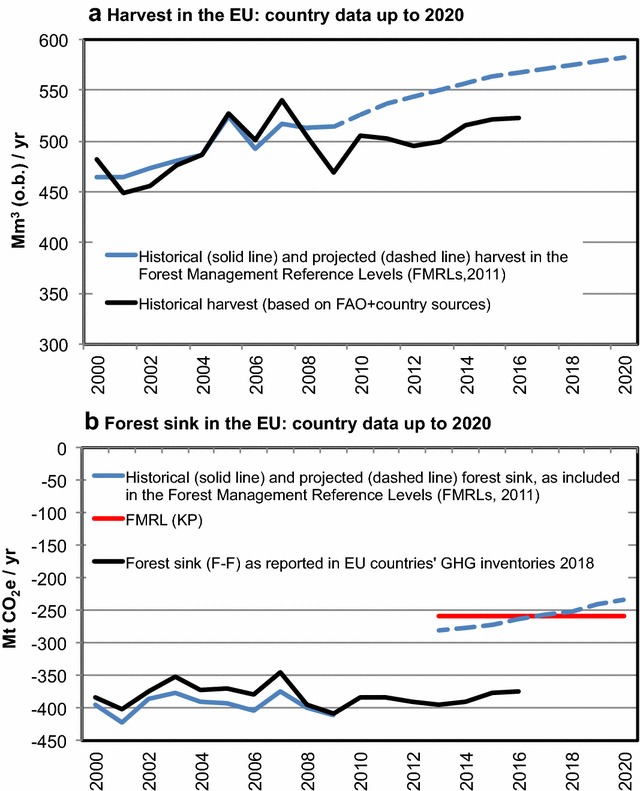
Comparison of historical and projected harvest (**a**) and forest sink without harvested wood products (**b**) as included in the forest management reference level (FMRL) submitted under the KP by EU countries in 2011 (blue lines) vs. analyses based on recent country data (black lines: FAO and other country statistics for harvest in **a**), 2018 GHG inventories for the sink in ‘forest remaining forest’ (F–F) up to 2016 in **b**. The red line in **b** is the sum of EU countries’ FMRLs (average for 2013–2020). Since ‘forest management’ (FM) is reported under KP only after 2013, to have a longer time series in **b** we used F–F for 1990–2016 (from 2018 GHG inventories) as proxy for FM. Following [10], the sink is expressed as negative number, i.e. more negative means a greater sink

The data reported by EU countries for the period 2013–2016 shows that the observed annual harvest at EU level was significantly lower (about 45 Mm^3^/year less) than that projected in FMRLs (Fig. 1a). This discrepancy may be explained by various factors, including an under-estimation of the impact of the 2009 economic crisis, and other factors that mean policies to increase harvest had not been implemented. This difference in projected vs. actual harvest, in turn, led to a reported forest sink in the 2018 GHG inventories that is much greater than the projected FMRL sink (Fig. 1b).

Using the available information on HWP and on “technical corrections” (i.e. corrections to be applied when accounting, to ensure methodological consistency [33]), the amount of forest credits at EU level would be about 110–120 Mt CO_2_/year (or about 70–80 Mt CO_2_/year, equivalent to 1.3% of 1990 EU total emissions, when the “3.5% cap” on credits is applied). These estimates are preliminary, because they are based only on the first 4 years out of the eight of the KP-CP2.

### Impact of the proposed approach on the expected harvest and sink in the EU

Based on the lessons learnt under the Kyoto Protocol, we developed an approach for a more credible accounting of the forest sink mitigation. Our approach is based on the principle that the accounting of mitigation outcomes should reflect fully the impact of changes in forest management practice (policy- or market-driven) relative to a historical reference period, similarly to the way GHG emissions are treated in other sectors. As a consequence, we propose that forest reference levels are projected assuming the business-as-usual “continuation of documented historical forest management practice”. This approach considers the country-specific forest characteristics and the forest age-related dynamic, but does not include assumptions on the future impact of policies (see “Methods” for details).

**Fig. 2:**
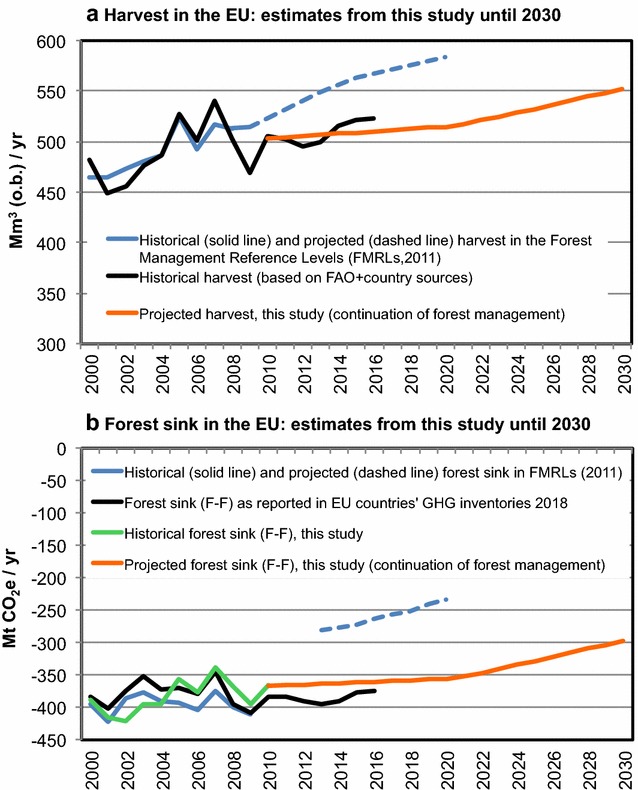
Comparison of harvest (**a**) and forest sink without Harvested Wood Products (**b**) estimated by EU countries for the historical period and projected until 2020 (blue and black lines, as shown in Fig. 1), with those estimated in this study for 2009–2030 (orange lines) based on the continuation of forest management practice documented for the period 2000–2009. The historical 2000–2009 sink estimated in this study is also shown in **b** (green line, “calibrated” with GHG inventory)

We estimated the harvest (Fig. 2a) and the forest sink (Fig. 2b) at EU level for the period 2009–2030, assuming the continuation of the forest management practice documented for the historical reference period (RP) 2000–2009.

**Fig. 3:**
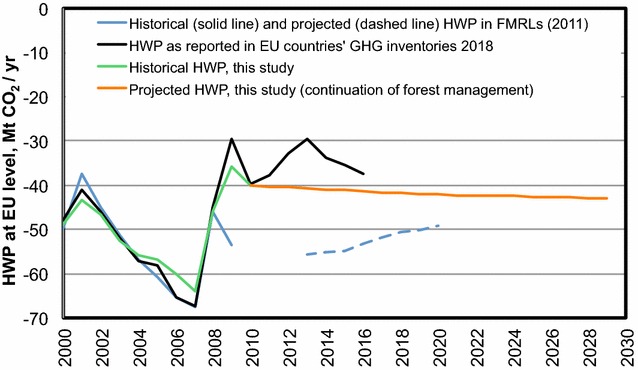
Comparison of historical and projected emissions and removals (net sink) from harvested wood products (HWP), as included in the forest management reference level (FMRL) submitted under the KP by EU countries in 2011 (blue lines) vs. 2000–2016 country HWP data from 2018 GHG inventories (black line), the historical 2000–2009 HWP data estimated by this study (green line) and the HWP estimated for 2009–2030 under the continuation of current forest management practice (orange line)

The historical and future evolution of net emissions from harvested wood products (HWP, Fig. 3) reflects the balance between the carbon inflow (affected by the current harvest) and the outflow (affected, among other things, by the long-term turnover rate of HWP commodities, influenced by past harvest rates). The influence of the inflow is evident comparing Figs. 2a and 3: the rapid increase in harvest observed between 2000 and 2007, followed by a rapid decline in 2008–2009 (Fig. 2a), is also partly reflected in the historical HWP trend (Fig. 3). Our estimated continuation of historical management practice produced a trend of a slightly increasing HWP “sink” up to 2030 (Fig. 3), because of the increased inflow associated with increased harvest.

**Fig. 4:**
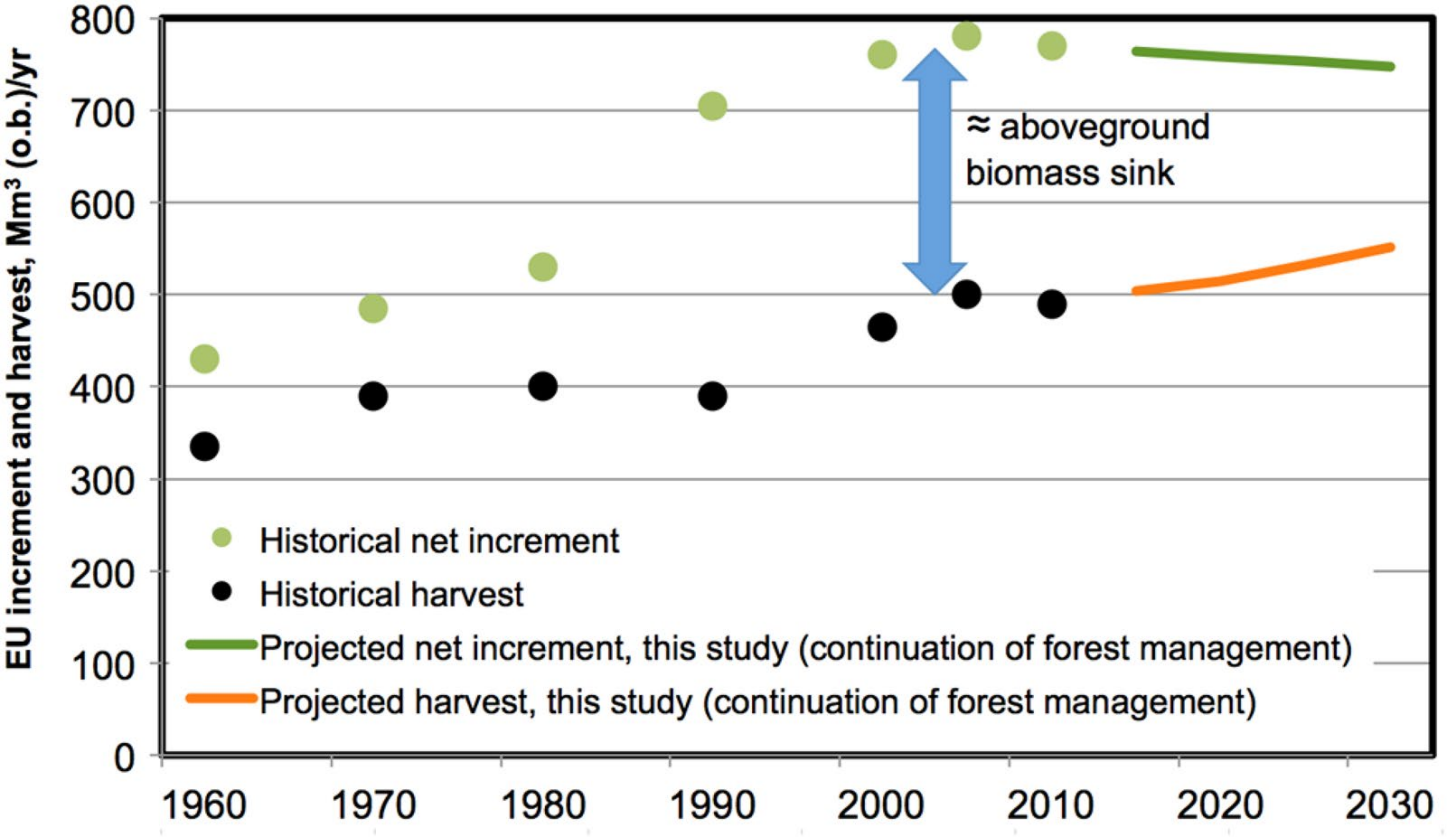
Comparison of forest net annual increment (implicitly including natural disturbances) and harvest at EU level for the historical period (dots, from [26]) with the values projected in this study up to 2030 (solid lines) following the continuation of forest management practice documented during 2000–2009. The blue arrow approximately represents the net aboveground biomass sink, i.e. the difference between net increment and harvest

Figure 4 shows the long-term evolution (1960–2010) of the historical net forest increment (rate of annual growth) and harvest at EU level (based on [26]), alongside our estimates of future increment and harvest expected up to 2030 assuming the continuation of historical forest management practice. Our projections suggest a slight decline in the net increment, consistent with the recent trend reported by EU forest inventories and in the scientific literature (e.g. [26]): after a long-lasting increase in net forest increment from 1960s to early 2000s, from around 2005 the forest increment at EU level showed the first signs of saturation and possible slight decline. It should be noted that our simulations do not incorporate the impact of environmental change (e.g. temperature, CO_2_), the effects of which have been a net sink in northern temperate regions during recent decades [34]. Since the biomass available for wood supply is expected to increase in the future (due to forest aging), application of our method means the absolute harvest volumes are also going up. This means that at the EU level, based on our results for the scenario of continuation of historical forest management practice, the proportion of harvest to net increment (i.e. the % of net increment that can be harvested as part of the reference level, i.e. without debits) is expected to increase by more than 10% in 2021–2030 relative to 2000–2009.

## Discussion

### A science-based approach for accounting the forest sink mitigation

The approach that we propose is based on the principle that the accounting of forest mitigation outcomes should reflect fully the impact of changes in forest management practice relative to a historical reference period. This allows the accounting of forest mitigation to be more comparable to other GHG sectors, and thus more credible, because all sectors implicitly reflect the impact of policy/management changes relative to a base year or period. This is a key feature of economy-wide climate targets under the Paris Agreement, where “one ton of carbon” in one sector should correspond to “one ton of carbon” in other sectors.

For instance, for a given area the emissions from the Agriculture sector depend, among other things, on the management of agricultural soils (e.g. on the amount of nitrogen fertilization). If this management remains constant, the associated emissions also remain constant. If some management practice changes, emissions also change (relative to a base year), and the reporting and accounting will reflect the emissions including the change.

For the areas of existing forests (“forest remaining forest”) the age-related dynamics complicate things for two reasons. First, growth rates are age dependent, and the age-class distribution of a landscape, which reflects past natural and human disturbances, therefore affects future growth. Second, the current forest management may be, e.g., to harvest a certain forest species at 80 years. The total amount of future harvest (i.e., a key driver of forest net emissions) does not only depend on the age at which harvest occurs, but also on the amount of forest area which reaches 80 years in a given period, i.e. on long-term legacy effects generated by past management and natural disturbances. One may continue the same management (e.g., harvest at 80 years) but the total amount of harvest over time will increase or decrease depending on the age structure of the forests. As a consequence, measuring the forest mitigation performance relative to the absolute emissions and removals of the base year (or period) may lead to outcomes that reflect age-related legacy effects rather than changes in forest management, with accounted credits and debits therefore not reflecting mitigation efforts.

We address this challenge by proposing to account the forest sink mitigation as deviation from a projected “forest reference level” (FRL) estimated assuming the “continuation of documented historical forest management practice”. This approach is based on three key concepts.

First, the method reflects continuation of management practice that is documentable, quantifiable and reviewable for a historical Reference Period (RP) comparable to the base year used in other GHG sectors. Forest management practice may be defined in different ways, based on country-specific circumstances. This typically includes the operations aimed at fulfilling specific forest functions (production, protection, etc.), such as the regeneration modality (natural or artificial) and the schedule and intensity of thinnings and final cut (e.g. [35]). Our approach simply requires identifying and documenting the country-specific forest management practices in the RP by using the best available data and quantifiable country-defined operational criteria (e.g., age, diameter, volume, etc.).

Second, the projection fully reflects the country-specific age-related forestry dynamics. To this aim, the continuation of forest management practice is combined with the expected changes in forest characteristics (e.g. biomass available for wood supply, net increment) as estimated deterministically from age-related dynamics after the RP.

Third, the projection does not include the assumed impact that existing or future policies and markets (i.e. demand-side dynamics) may have on future forest management practices. This represents the main difference with the approach under the Kyoto Protocol, where the assumed future impact of pre-2009 policies on the projected forest management was implicitly allowed. However, our approach will inherently reflect—through the documentation of management practice during the RP—the already observed impact of policies and markets enacted during the RP.

Therefore, our approach is flexible to accommodate country-specific circumstances yet is science-based, because it builds on documentable and reviewable past management practices (and does not include unreviewable assumptions on the future impacts of policies). The main aim of our approach is to enable a scientifically robust, transparent and credible accounting of forest mitigation, making it more similar to the way GHG emissions are treated in other sectors, while avoiding potentially “unfair” outcomes associated with the possible projected decline of the forest sink or increase of forest harvest because of age-related dynamics. At the same time, our approach maintains the appropriate incentives/disincentives for beneficial/detrimental actions, i.e. the positive or negative atmospheric impact of changes in management relative to the historical period are fully reflected in the accounts.

While the concepts above are particularly relevant for the EU, because of the predominant role of age structure dynamics in determining EU forest GHG fluxes, our approach is potentially applicable to all countries.

Building on an earlier methodological report [30] supporting the EU legislative LULUCF proposal [28], here we illustrate and implement at EU level a method aimed at implementing the principle above. Our method helps to distinguish between a change in harvest rate that results from policy changes, from a change in harvest that is independent of policy changes (see Methods for details). When implementing our method at EU level, here we used 2000–2009 as the RP. This RP ensures a good comparability with other GHG sectors (whose targets are expressed relative to 2005) and excludes the impact of national policies that, following the adoption of the 2009 EU renewable energy directive, incentivized forest bioenergy and thus increased wood demand.

The refined and more detailed approach and calculations presented in this paper should help clarifying some common misunderstanding of the approach as presented earlier [30] and widely debated: First, the forest reference that we propose is not “an estimate of the average annual net emissions or removals realized in the past” (e.g. [36]), but incorporates fully the impact of age-related forest dynamics in future emissions and removals. Second, arguing that the continuation of historical forest management practices would lead to chose “incorrect harvesting strategies” [36] implies that our approach aims to identifying uniquely the best harvesting strategy (for the propose of climate mitigation), which is not the case. The best forest mitigation strategy is the one that optimizes the sum of all mitigation options in a given (policy-determined) time frame, a complex task whose solution is very much country-specific (e.g., [6, 37]). If such country-specific solutions are identified and implemented, any resulting reductions in emissions or increases in sinks relative to the reference level will be accounted by countries. Therefore, our approach implicitly encourages those improvements to forest management that improve the net GHG balance.

We note that other methods may exist that fulfill the principles above. For example, if a country has very precise information on the forest management practice that actually occurred during the historical RP, then the country may directly use this information, without necessarily performing all the specific calculations described here. Instead, our method has been specifically designed to be potentially also applicable when only generic information on historical management practice is available, which is the most likely situation.

Our method, as any modeled projection, contains uncertainties, mainly related to the original input data and to methodological assumptions. Different factors, such as initial age class distribution (i.e., at the beginning of the model run), past natural disturbances (fires and storms), the criteria and timing for thinnings and final cuts, the share of harvest between different silvicultural operations and between different species, may considerably affect the projected age class distribution and, as a consequence, the future amount of harvest [38]. Other sources of uncertainty are the future impact of natural disturbances [39] and of climate change or atmospheric CO_2_ [36], not addressed in our study.

### Why including policies in reference levels undermines the credibility of the accounting

The analysis presented here supports and reinforces previous suggestions (e.g. [16, 21, 23, 40, 41]), i.e. that including policy-driven harvest assumptions in the FRL risks compromising the accuracy and credibility of the forest accounting, as summarized and further developed in the following points.

- Risk of “”windfall” credits, i.e. credits for no activity: Based on the available data (Fig. 1), we show that the forest management sink reported at EU level for the first 4 years of the KP-CP2 (i.e. 2013–2016) would result in potential credits equal to about 110–120 Mt CO_2_/year (or 70–80 Mt CO_2_/year, with the cap on credits applied, equivalent to about 1.3% of 1990 EU total emissions). Our estimates based on the continuation of forest management practice documented during 2000–2009 (Fig. 2) suggest that most of these potential KP-CP2 forest credits do not reflect a genuine change in management, but are rather associated with the high projected harvest rates assumed at the time of setting the reference level (2011), and which have in fact not yet materialized. Although this analysis is preliminary, it raises legitimate doubts on the credibility of forest “credits” accounted as a result of deviations from policy assumptions that are essentially not reviewable from a technical point of view (a review of policy assumption may be perceived as a policy judgment, not acceptable under a review process, e.g. [19]). Adding a cut-off date on policies (e.g. 2009, as for KP-CP2) does not necessarily help, because policies approved before that date (e.g. plans of future new biomass power plants) may still potentially justify including a policy-assumed future increase of harvest in the reference level.
- Risk of “hiding emissions”, i.e. omitting policy-driven increases in emissions: From an atmospheric perspective, a reduction in the forest sink leads to more CO_2_ remaining in the atmosphere and is thus mathematically equivalent to a net increase in emissions. If this reduction in sink is driven by policy-related harvest increases, including it in the FRL means effectively “hiding” the impact of new or modified forest policies on resulting changes in forest management from the accounts. No other GHG sector is allowed to omit anthropogenic emissions from accounting. Even under “sustainable” forest management, e.g., when harvest does not exceed forest growth (so the forest carbon stock does not decline), omitting a policy-driven decrease of the sink from the accounts cannot be justified if credibility and comparability with other sectors is to be maintained. Should this be allowed, in the EU a loss of a sink of up to 380 Mt CO_2_/year (i.e. the current sink in forest remaining forest) could be “seen by the atmosphere” but disappear from the accounts. This issue of credibility and comparability holds true even if the policies behind the harvest increase are well justifiable from other perspectives (adaptation, bio-economy, stimulation of future sink, etc.).

Both points above are linked to the cross-sectorial consistency and comparability. Higher harvest rates may reduce the forest sink, but use of the extra harvested wood may lead to increased carbon stored in wood products and extra emission reductions in other sectors, e.g. through the substitution of wood for other more emissions-intensive materials (e.g. cement) or fossil fuels for energy purposes. Regardless of whether or not these emission reductions in other sectors fully compensate the reduced LULUCF sink due to extra harvest, they will be implicitly fully counted in the non-LULUCF sectors. With specific regard to bioenergy, the IPCC guidance [10] does not assume that bioenergy use is “carbon neutral” (i.e. that biomass combustion emissions are always compensated by regrowth), but that any carbon loss is reported (and implicitly accounted) under the LULUCF sector rather than under the energy sector, to avoid double counting. Including policies in the FRL (e.g., policies incentivizing forest bioenergy, which leads to increased wood demand) means factoring out the impact of such policies from the accounting. This would undermine the comparability with the other economic sectors, where the atmospheric impact (positive or negative) of any policy after the base year is fully reflected in the accounts. Therefore, to avoid bias through incomplete accounting, and to ensure consistency and comparability with other GHG sectors, the full impact of policy-based changes in harvest beyond the continuation of management practices should be accounted for in the LULUCF sector.

### Implications for the EU

There has recently been much debate within the EU on the proposed legislation for including LULUCF in the EU 2030 climate target [28]. On the most controversial topic, i.e. on how to account forest mitigation through projected reference levels [29], the approach described here is compatible with the final EU LULUCF regulation [42].

The implementation of our approach at EU level shows that harvest volumes are expected to increase by 9% in the period 2021–2030, relative to 2000–2009, with a consequent reduction of the sink (by about 15%). This increase in harvest is slower than that assumed under the Kyoto Protocol (for 2013–2020) and it reflects only age-related dynamics, i.e. the increased growing stocks in maturing forests require more harvest to continue the forest management practice documented historically. This extra harvest will in any case provide opportunities for additional mitigation through material and energy substitution, without generating “debits” against the reference level. On the other hand, the impact of actual deviations from the historical management practice will be reflected in the account, like in any other GHG sector.

The decrease of the sink associated with our projected increase in harvest may be actually lower than we estimated. This is because our model runs did not include the impact of climate change or atmospheric CO_2_ on forest growth, which at EU level is likely to enhance growth [34] (especially in Nordic countries [36])—although considerable uncertainty exists on the impact of natural disturbances [39],—and because large opportunities exist to enhance forest growth through new management practices [43], beyond the business-as-usual continuation of historical management practice that we considered.

### Implications for bioenergy accounting

The EU legislation on bioenergy [44] mirrors international (IPCC) rules and relies on the fact that carbon emissions are fully accounted under LULUCF in each country from which the biomass originates. Where the LULUCF sector is included in the economy-wide and internationally agreed commitments (as for the EU), if emissions occur in the LULUCF sector from biomass used for energy, they would have to be compensated by emission reductions elsewhere in the economy [45]. In this context, we think that our proposed approach on FRL will help reconcile the very polarized debate on the use of forest bioenergy (e.g. [46, 47]). As noted by [48], there are strong reasons to object to generalized statements on the climate effect of forest bioenergy. While an in depth analysis of the climate effects of forest bioenergy is outside the scope of this paper, we do note that our approach is policy-neutral: it does not assume a priori that increasing bioenergy is good or bad, but requires the atmospheric impact of any bioenergy use associated with changes in management to be fully reflected in the country LULUCF accounts. In that respect, our approach leaves entirely to the countries the evaluation of which mix of forest mitigation options (e.g., increasing the forest sink, increasing carbon storage in harvested wood products, or increasing energy and/or material substitution) is more effective in their specific circumstances. At the same time, our approach will represent, if implemented, a strong incentive for countries to promote those forms of wood uses and bioenergy (e.g., including the cascading use of wood [49]) whose impacts effectively reduce net GHG emissions, and discourage those which result in negative impacts on the atmosphere (e.g. [50]).

### Implications for the Paris Agreement

The long-term goal of the Paris Agreement cannot be reached without a substantial and credible contribution from forests. Therefore, countries “should take action to conserve and enhance sinks”, and “shall promote environmental integrity, transparency, accuracy, completeness, comparability and consistency” in accounting towards their NDCs. While the forest sink can contribute to GHG emission reductions in many countries [2, 3, 6], the credibility of this option is often challenged. In the context of a possible lack of precise rules on forest accounting under the Paris Agreement, the approach proposed here, compatible with the new EU legislation, may represent a precedent that helps other countries to make the forest sector more comparable to other GHG sectors, and therefore supports the much-needed credibility of the forest sink mitigation [2].

## Conclusions

For the economy-wide country climate targets under the Paris Agreement to be credible, the accounts should reflect the atmospheric impact of all the changes in management activities (policy- or market-driven) relative to a base year (or baseline). This is challenging for the forest sector, because age-related legacy effects associated with past management and natural disturbances affect future net emissions. A way to address this challenge is accounting future mitigation as deviation from a projected (forward-looking) “forest reference level”. Under the Kyoto Protocol (2013–2020), these reference levels considered age-related forest dynamics, but also implicitly allowed the inclusion of the assumed future implementation of approved policies.

We show why including policy assumptions in reference levels undermines the credibility of the accounting. Our analysis of provisional results (for 2013–2016) for the EU forest reference levels under the Kyoto Protocol indicates that most of the anticipated 110–120 Mt CO_2_/year of forest credits (capped at 70–80 Mt CO_2_/year, equal to about 1.3% of 1990 EU total emissions) do not reflect real mitigation actions but mostly deviations from policy-assumed increases of harvest that failed to materialise. Conversely, had these policies materialized, a policy-driven reduction in the EU forest sink (equivalent to an increase in net emissions) would have been omitted from the accounts. This is not comparable with the way emissions are treated in other GHG sectors.

Instead we propose a science-based framework to assess the atmospheric impact of forest mitigation actions in the context of country climate targets. The main aim of our approach is to enable a transparent and credible accounting of forest mitigation, making it more similar to the way GHG emissions are treated in other sectors, while avoiding potentially unfair outcomes associated with the possible projected decline of the forest sink or increase of forest harvest because of age-related dynamics. To this aim, forest reference levels are projected assuming the continuation of historically documented forest management practices. This approach does not include assumptions on the future impact of policies but considers fully the country-specific forest characteristics and the age-related forest dynamics, i.e. depending on the age-class legacy resulting from past management and natural disturbances, continuation of historical forest management activities may lead to future increases or decreases in the carbon sink. As a result, countries are not “penalized” if forests get older, or past management successes result in increased available timber volumes in the future. The approach described here is compatible with the EU Regulation including the forest sector in the EU 2030 climate targets [42].

We then illustrate, and apply at EU level, a possible method to implement this approach. Our results shows that, because of increased timber volumes resulting from aging forests in many EU countries, the continuation of historical forest management practice implies increasing harvest rates by about 12% in 2030 at EU level, relative to a historical reference period 2000–2009 (Fig. 2). This extra harvest, and the consequent reduction of the sink, are associated with age-related dynamics and not with policy changes, and therefore will not generate “debits” against the reference level.

Our proposed approach offers a credible solution to the controversial debate on accounting the forest sink at the country level, particularly polarized in the case of forest bioenergy, and helps to increase the transparency and scientific credibility of forest mitigation within the Paris Agreement.

## Methods

### Retrospective analysis of reference levels under the Kyoto Protocol: the EU case

We analyzed the impact of the Forest Management Reference Level (FMRL) used for the KP-CP2 on the potential accounting credits from ‘forest management’ (FM) at EU level, for the period 2013–2016. This analysis is preliminary, because only the first 4 years of the eight of the KP-CP2 are assessed. We compared the values of projected harvest and sink included in the EU countries’ FMRL submissions (2011) with recent published data on actual values, i.e. FAO and other country statistics for the harvest [38], and the 2018 GHG inventories for the sink in the ‘forest remaining forest’ (F–F) category [25]. To facilitate the comparison and have a longer time series, here we use F–F (as reported in the 2018 GHG inventories for 1990–2016) as proxy for FM (as reported under KP only for 2013–2016); although in specific countries F–F and FM may slightly differ for the years 2013–2016 (F–F includes the area being forest for at least 20 years, while FM includes the area being forest since 1990), the difference at the EU level is negligible (< 1%).

The data on F–F sink were complemented by the available information (from 2018 GHG inventories) on harvested wood products (HWP) and on “technical corrections” (i.e. corrections to ensure methodological consistency between the FMRL and reported GHG estimates [33]), in order to obtain a preliminary estimate of the potential FM credits at EU level for the period 2013–2016, with or without the “cap” of forest credits.

### Proposed principle to project business-as-usual forest management

The conceptual framework that we propose for accounting mitigation from forest management is based on the principle that the accounting of mitigation outcomes should reflect fully the impact of changes in forest management practice (policy- or market-driven) relative to a historical reference period. This principle makes the forest accounting comparable to other GHG sectors.

The approach that we propose aims to fulfill the above principle through a “forest reference level” (FRL) based on projected business-as-usual continuation of historical management practice, i.e. continuing the forest management practice documented in a historical Reference Period (RP). This RP is comparable to the base year used in other GHG sectors.

This approach builds on documentable and reviewable past management practices (that should be defined by the country), fully reflects the country-specific age-related forestry dynamics, and does not include unreviewable assumptions about the future impacts of policies. In other words, our approach is based on the supply-side deterministic evolution of forest resources, but ignores the demand-side dynamics (i.e. possible future impact of policies and markets).

The principle and concepts above may be implemented with different methods. For instance, if a country has very precise information on the forest management practice that actually occurred during the historical RP, based on model reconstructions and/or silvicultural management plans and thinning and harvest records for individual stands (e.g., for even-aged Norway spruce privately owned, final clear-cut occurs at 90 years and thinning of 20% of biomass occurs at 25, 40 and 55 years, etc.), the model may directly use this information. In this case, which is very data intensive, the harvest would be an output of the model. However, a second case is more likely, i.e. that information on management practice during the RP can be expressed only through ranges, based on plans, silvicultural books or expert judgment (e.g. for even-aged Norway spruce privately owned, final clear-cut occurs between 80 and 140 years and thinning occurs anywhere between 20 and 60 years). For this latter case (information on management practice expressed as ranges), we developed and implemented at EU level a possible (i.e., non-exclusive) method to implement the principle outlined above.

In the following two sections, we first illustrate the key steps of this method, largely following an earlier methodological report [30] supporting the EU LULUCF legislation, and then we describe the implementation of this method at EU level with the Carbon Budget Model.

### Illustrative methodological steps to implement the proposed principle

The purpose of this section is to illustrate the key methodological steps applied to produce the results shown in the next sections. For further details, see [30].

**Fig. 5:**
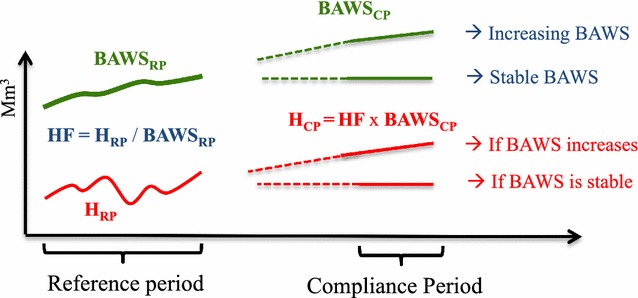
Conceptual illustration of “biomass available for wood supply” (BAWS) and harvest volumes (H) during the historical reference period (RP), and possible evolution during the future compliance period (CP). The historical BAWS and H are used to calculate the harvest fraction (HF) during the RP, for each stratum and management practice. This HF is then multiplied by the estimated future evolution of BAWS in the CP, to obtain the future harvest in the CP expected with the continuation of historical forest management practice. Note that, if the forest is getting older, the BAWS_CP_ will typically increase relative to the RP and, as a consequence, H_CP_ will also increase. See text for details

- Step 1. Stratify the area of “forest remaining forest” (F–F), based on national circumstances and data availability. Each stratum is typically characterized by specific management objectives and supporting practices which may depend, among other things (Duncker et al. [35]), on (i) predetermined (and largely un-modifiable) conditions, such as the climate and bio-geophysical site conditions; (ii) the forest species/type, and (iii) the functions assigned to a certain forest area (production, protection, recreation, etc.), affected also by the ownership.
- Step 2. Identify and document the forest management practices for each stratum during the RP, using the best available data. Each management practice (e.g. thinning and final cut) is described through quantifiable country-defined “operational criteria” (e.g., age, diameter or volume at which thinning or final harvest occurs) representing the most plausible estimate of the practices applied during the RP. For example, an even-aged conifer high forest (i.e., a forest originated from seed or from planted seedlings), whose main function is timber production, may require a clearcut between 60 and 100 years, while an uneven-aged mixed forest requires partial or selective cutting.
- Step 3. Project the evolution of F–F area. This area may change in time due to two dynamic processes, i.e. area of “land converted to forest” entering the F–F category (after a transition period, typically 20 years), and area of F–F converted to other land-uses (i.e. deforestation). While for the gross expansion of F–F area data from GHG inventories can be used (i.e. the area of land converted to forest in the period 2001–2005 is typically expected to enter the F–F category in 2021–2025), for deforestation it can be assumed that the past rate of deforestation (as documented in the country GHG inventory for the RP) will continue.
- Step 4. Project the future carbon gains (step 4.1, forest increment) and losses (step 4.2, i.e., harvest, mortality, natural disturbances) in each forest carbon pool and stratum of F–F, and then project the carbon stock change in harvested wood products pool (HWP, step 4.3).
  - Step 4.1. The forest increment is calculated by combining, for each stratum, the expected evolution of increment (i.e. as affected by age structure and yield curves) with the continuation of the management practices described in step 2. Iterations with step 4.2 may be needed.
  - Step 4.2. Here we summarize the procedure to calculate the carbon losses due to future harvest expected under the continuation of the management practices (for other losses and non-CO_2_ emissions, see [30]). For each stratum and management practice, the following sub-steps need to be implemented (see Fig. 5).

    - *Calculate the* “*biomass available for wood supply*” during the historical RP, BAWS_RP_ (including wood for energy uses). This BAWS is the potential biomass subject to each operational criterion defined above for each forest management practice and in each stratum (e.g. if 80–140 years is the range at which final cut occurred for Norway spruce during the RP, the BAWS is the biomass available in this range). Each stratum can be potentially subject to multiple operations (e.g. thinning and final felling, can occur in the same stratum, on different age classes).
    - *Document the harvest volumes* (e.g., m^3^) during the historical RP (H_RP_), based on statistics and/or modelling analysis.
    - *Calculate the harvest fraction* (HF_RP_, as average for the RP) as:
      1 $$HF_{RP} = \frac{{\overline{{HF_{RP} }} }}{{\overline{{BAWS_{RP} }} }}$$
      The HF_RP_ is a proxy that implicitly expresses the impact of all constraints (markets, policies, owners’ behavior, accessibility, etc.) on harvest volumes during RP. H_RP_ BAWS_RP_ are, respectively, harvest volumes and biomass available for wood supply for the historical RP. This parameter provides a link between the broadly defined forest management practice (e.g. through ranges) and the amount of harvest that actually occurred during the RP.
    - *Calculate the expected evolution of the biomass available for wood supply in the compliance period* (CP, i.e. when accounting will occur), BAWS_CP_, by applying the same management practices of the RP (e.g., clearcut between 60 and 100 years) to the expected age-related evolution of forest characteristics (e.g., biomass, increment).
    - *Calculate the future harvest* during the CP *(H*_*CP*_*)*, by multiplying the historical harvest fraction (Eq. 1) by the expected biomass available in the CP (BAWS_CP_), for each stratum and management practice:
      2 $$H_{CP} = HF_{RP} \;*\;BAWS_{CP}$$
  - Step 4.3. For the HWP pool, assuming the continuation of the IPCC methodologies for the “production” approach [33], the following data and assumptions can be used (based on [51]):
    - Project the *amount* of wood commodities entering the HWP pool in the CP consistently with the estimated harvest level during the CP, by assuming the use of the same fraction of harvest for the HWP commodity production as in the RP. This implicitly means continuing with the same % share of energy vs. non-energy use of wood as documented for the historical RP.
    - Project the *use* of wood in the CP by using the same % of HWP commodities (sawnwood, wood-based panels, paper and paperboard) as documented for the RP.

Once all the components above have been estimated, the projections of CO_2_ emissions and removals associated with the continuation of the management practices in F–F may be calculated as the sum of all gains and losses for all strata and years in the CP.

### Implementation of the proposed method at EU level

The method above was applied to 26 EU countries (all EU countries except Malta and Cyprus), using the Carbon Budget Model (CBM) developed by the Canadian Forest Service [31].

The CBM is an inventory-based, yield-curve-driven model that simulates the stand- and landscape-level C dynamics of above- and belowground biomass, dead organic matter (DOM; litter and dead wood) and mineral soil. The model has been already implemented at the EU level to estimate the forest C dynamics from 2000 to 2012 [52] and the future carbon budget and fluxes under different management scenarios to 2030 [38]. The main input data come from National Forest Inventories (NFIs, see [30, 38, 53]). Here we apply the same methods, data and assumptions used in these studies. The spatial framework applied by the CBM conceptually follows IPCC reporting method 1 [10], in which the spatial units are defined by their geographic boundaries and all forest stands are geographically referenced to a spatial unit (SPU). The intersection between 26 administrative units (i.e., European countries) and 36 climatic units yielded 910 SPUs. Within a SPU, each forest stand is characterized by age, area and seven classifiers that provide administrative and ecological information: the link to the appropriate yield curves; the parameters defining the silvicultural system, such as the forest composition (defined according to different forest types, FTs) and the management type (MT). From the NFIs of each country, we derived (i) the country-specific original age-class distribution (for the even-aged forests), (ii) the main FTs based on the forest composition, (iii) the average volume and current annual increment (if possible, defined for each FT), and (iv) the main MTs. The MT parameters may include even-aged high forests, uneven-aged high forests, coppices and specific silvicultural systems such as clear cuts (with different rotation lengths for each FT), thinnings, shelterwood systems, partial cuttings, etc. In a few cases, because of the lack of country-specific information, some of these parameters were derived either from the literature or from average values reported for other countries. Additional methodological details and country-level input data may be found in [32, 52, 54].

Consistently with the EU LULUCF Regulation [42], the implementation of our method at EU level used 2000–2009 as the RP.

The country-specific stratification, the forest management practices and their associated quantitative operational criteria (steps 1 and 2 above) were defined according to information provided by the countries, found in the literature or through an expert assessment (see [30, 53] for a summary of country data sources). The main operational criterion used in our study was the minimum rotation age, except for thinnings and uneven-aged forests where the minimum time interval between two consecutive operations has been applied. The evolution of F–F area in our model runs used data from country GHG inventories, as described in step 3 above.

With regards to the calculation of carbon gains and losses in various pools (step 4 above), the links between living biomass, dead wood, litter and mineral soil are automatically modelled by the CBM [31]. The model runs shown here do not include the impact of climate change or atmospheric CO_2_ and nitrogen fertilization on forest growth. While our model runs took into account the impact of all major historical natural disturbances [54], no disturbances have been assumed after the RP. Other carbon losses (e.g. mortality) occurring after the RP were automatically included by the CBM model [31, 32]. Input data and methods applied to estimate the HWP emissions and removals for the RP are described in [55].

Since a model used to project the FRL should be able to reproduce historical data from the national GHG inventory [33], the GHG emissions and removals estimated by CBM after 2000 were “calibrated” (i.e., adjusted ex-post) to match the historical emissions and removals data in F–F, as reported by the 2018 GHG inventories for the period 2000–2009. This procedure, identical to the one applied by many EU countries when setting the FMRL under the Kyoto Protocol, represents an application of the ‘overlap’ method [10, 33] to ensure time-series consistency when different methods are used over time. This procedure does affect the projected trend. The magnitude of the calibration carried out on our results (i.e., the difference between the original CBM results and the GHG inventories for the period 2000–2009) is significant for some EU countries, but is small at the EU level. The average EU-level 2000–2009 sink is − 380 Mt CO_2_/year based on GHG inventories and − 396 Mt CO_2_/year based on the CBM runs; therefore, the original CBM results were corrected with + 16 Mt CO_2_/year for the whole time series.

## Abbreviations

LULUCF: land use, land-use change and forestry
FRL: forest reference level
FMRL: forest management reference level (under the Kyoto Protocol)
F–F: forest remaining forest (forest since at least 20 years)
RP: reference period
CP: compliance period
HWP: harvested wood products
HF: harvest fraction
BAWS: biomass available for wood supply

## References

[1] UNFCCC. Adoption of the Paris Agreement. Report No. FCCC/CP/2015/L.9/Rev.1. 2015. https://unfccc.int/resource/docs/2015/cop21/eng/l09r01.pdf. Accessed 11 May 2018.

[2] Grassi G, House J, Dentener F, Federici S, den Elzen M, Penman J (2017). The key role of forests in meeting climate targets requires science for credible mitigation. Nat Clim Chang.

[3] Griscom BW, Adams J, Ellis PW, Houghton RA, Lomax G, Miteva DA (2017). Natural climate solutions. Proc Natl Acad Sci USA.

[4] Rockström J, Gaffney O, Rogelj J, Meinshausen M, Nakicenovic N, Schellnhuber HJ (2017). A roadmap for rapid decarbonization. Science.

[5] Kurz WA, Smyth C, Lemprière T (2016). Climate change mitigation through forest sector activities: principles, potential and priorities1. Unasylva.

[6] Smyth CE, Stinson G, Neilson E, Lemprière TC, Hafer M, Rampley GJ (2014). Quantifying the biophysical climate change mitigation potential of Canada’s forest sector. Biogeosciences.

[7] Böttcher H, Kurz WA, Freibauer A (2008). Accounting of forest carbon sinks and sources under a future climate protocol-factoring out past disturbance and management effects on age-class structure. Environ Sci Policy.

[8] UNFCCC. LULUCF. http://unfccc.int/land_use_and_climate_change/lulucf/items/1084.php. Accessed 11 May 2018.

[9] Iversen P, Lee D, Rocha M, Canaveira P, Davis G, Elias P, et al. Understanding land use in the UNFCCC. 2014. http://www.climateandlandusealliance.org/wp-content/uploads/2015/08/Understanding_Land_Use_in_the_UNFCCC.pdf. Accessed 11 May 2018.

[10] IPCC. IPCC guidelines for national greenhouse gas inventories. In: Eggleston HS, et al., editors. National greenhouse gas inventories programme, institute for global environmental strategies. 2006. https://www.ipcc-nggip.iges.or.jp/public/2006gl/. Accessed 11 May 2018.

[11] IPCC, Eggleston S, Srivastava N, Tanabe K, Baasansuren J, editors. Revisiting the use of managed land as a proxy for estimating national anthropogenic emissions and removals. 2010. https://www.ipcc-nggip.iges.or.jp/public/mtdocs/pdfiles/0905_MLP_Report.pdf. Accessed 11 May 2018.

[12] Cowie AL, Kirschbaum MUF, Ward M (2007). Options for including all lands in a future greenhouse gas accounting framework. Environ Sci Policy.

[13] Canadell JG, Kirschbaum MUF, Kurz WA, Sanz MJ, Schlamadinger B, Yamagata Y (2007). Factoring out natural and indirect human effects on terrestrial carbon sources and sinks. Environ Sci Policy.

[14] Schlamadinger B, Bird N, Johns T, Brown S, Canadell J, Ciccarese L (2007). A synopsis of land use, land-use change and forestry (LULUCF) under the Kyoto Protocol and Marrakech Accords. Environ Sci Policy.

[15] Krug JHA (2018). Accounting of GHG emissions and removals from forest management: a long road from Kyoto to Paris. Carbon Balance Manag.

[16] Dooley K, Gupta A (2017). Governing by expertise: the contested politics of (accounting for) land-based mitigation in a new climate agreement. Int Environ Agreem Polit Law Econ.

[17] Kurz WA. “Large inter-annual variations in carbon emissions and removals”. IPCC. Revisiting the use of managed land as a proxy for estimating national anthropogenic emissions and removals. In: Meeting Report, 5–7 May, 2009, INPE, Sao Jose dos Campos, Brazil. 2010. https://www.ipcc-nggip.iges.or.jp/public/mtdocs/pdfiles/0905_MLP_Report.pdf. Accessed 11 May 2018.

[18] UNFCCC. Decision 2/CMP.7 land use, land-use change and forestry. https://unfccc.int/files/meetings/durban_nov_2011/decisions/application/pdf/awgkp_lulucf.pdf. Accessed 11 May 2018.

[19] UNFCCC. Decision 2/CMP.6 the Cancun agreements: land use, land-use change and forestry. R. 2011. p. 1–32. http://www.ciesin.columbia.edu/repository/entri/docs/cop/Kyoto_CMP6_dec02.pdf. Accessed 11 May 2018.

[20] UNFCCC. Technical assessments of the forest management reference level submissions. http://unfccc.int/bodies/awg-kp/items/5896.php. Accessed 11 May 2018.

[21] Macintosh AK (2012). LULUCF in the post-2012 regime: fixing the problems of the past?. Clim Policy.

[22] Grassi G, den Elzen MGJ, Hof AF, Pilli R, Federici S (2012). The role of the land use, land use change and forestry sector in achieving Annex I reduction pledges. Clim Change.

[23] Greenglass N, Funk J, Chaum M, Houghton RA (2010). Fixing a flawed approach to forest accounting in the next round of the Kyoto Protocol. Carbon Manag.

[24] Forest Europe. State of Europe’s forests. 2015. http://foresteurope.org/state-europes-forests-2015-report/. Accessed 11 May 2018.

[25] European Environment Agency. Annual European Union greenhouse gas inventory 1990–2015 and inventory report. 2017.

[26] Nabuurs G-J, Lindner M, Verkerk PJ, Gunia K, Deda P, Michalak R (2013). First signs of carbon sink saturation in European forest biomass. Nat Clim Chang.

[27] European Comission. EU reference scenario 2016. 2016. https://ec.europa.eu/energy/sites/ener/files/documents/ref2016_report_final-web.pdf. Accessed 11 May 2018.

[28] European Commission. Proposal for a regulation on the inclusion of greenhouse gas emissions and removals from land use, land use change and forestry into the 2030 climate and energy Framework, vol. 230; 2016.

[29] Euractive. LULUCF dossier. https://www.euractiv.com/topics/lulucf/. Accessed 11 May 2018.

[30] Grassi G, Pilli R (2017). Projecting the EU forest carbon net emissions in line with the “continuation of forest management”: the JRC method. Tech Rep.

[31] Kurz WA, Dymond CC, White TM, Stinson G, Shaw CH, Rampley GJ (2009). CBM-CFS3: a model of carbon-dynamics in forestry and land-use change implementing IPCC standards. Ecol Modell.

[32] Pilli R, Grassi G, Kurz WA, Smyth CE, Blujdea V (2013). Application of the CBM-CFS3 model to estimate Italy’s forest carbon budget, 1995–2020. Ecol Modell.

[33] IPCC, Hiraishi T, Krug T, Tanabe K, Srivastava N, Baasansuren J, Fukuda M, et al., editors. IPCC 2013 revised supplementary methods and good practice guidance arising from the Kyoto Protocol task force on national GHG inventories. 2014. p. 1–14. https://www.ipcc-nggip.iges.or.jp/public/kpsg/pdf/KP_Supplement_Entire_Report.pdf. Accessed 11 May 2018.

[34] Sitch S, Friedlingstein P, Gruber N, Jones SD, Murray-Tortarolo G, Ahlström A (2015). Recent trends and drivers of regional sources and sinks of carbon dioxide. Biogeosciences..

[35] Duncker PS, Barreiro SM, Hengeveld GM, Lind T, Mason WL, Ambrozy S, Spiecker H (2012). Classification of forest management approaches: a new conceptual framework and its applicability to European forestry. Ecol Soc.

[36] Vauhkonen J, Packalen T (2018). Uncertainties related to climate change and forest management with implications on climate regulation in Finland. Ecosyst Serv..

[37] Lundmark T, Bergh J, Hofer P, Lundström A, Nordin A, Poudel BC (2014). Potential roles of Swedish forestry in the context of climate change mitigation. Forests.

[38] Pilli R, Grassi G, Kurz WA, Fiorese G, Cescatti A (2017). The European forest sector: past and future carbon budget and fluxes under different management scenarios. Biogeosciences.

[39] Seidl R, Schelhaas MJ, Rammer W, Verkerk PJ (2014). Increasing forest disturbances in Europe and their impact on carbon storage. Nat Clim Chang..

[40] Grassi G. Robust and credible accounting rules for forests. http://forest.jrc.ec.europa.eu/activities/lulucf/presentations/. Accessed 11 May 2018.

[41] House J. Forest accounting rules put EU’s climate credibility at risk. https://www.euractiv.com/section/climate-environment/opinion/forest-accounting-rules-put-eus-climate-credibility-at-risk/. Accessed 11 May 2018.

[42] EU. LULUCF regulation. http://data.consilium.europa.eu/doc/document/ST-8049-2018-INIT/en/pdf, see item 9. Accessed 11 May 2018.

[43] Nabuurs GJ, Delacote P, Ellison D, Hanewinkel M, Hetemäki L, Lindner M (2017). By 2050 the mitigation effects of EU forests could nearly double through climate smart forestry. Forests.

[44] European Commission. Renewable energy directive. http://eur-lex.europa.eu/legal-content/EN/TXT/HTML/?uri=CELEX:52016PC0767&from=EN. Accessed 11 May 2018.

[45] European Commission. Impact assessment—sustainability of bioenergy accompanying the document proposal for a directive of the European parliament and of the council on the promotion of the use of energy from renewable sources. 2016. https://ec.europa.eu/energy/sites/ener/files/documents/1_en_impact_assessment_part4_v4_418.pdf. Accessed 11 May 2018.

[46] Euractiv. Bioenergy dossier. https://www.euractiv.com/topics/bioenergy/. Accessed 11 May 2018.

[47] Searchinger TD, Beringer T, Strong A (2017). Does the world have low-carbon bioenergy potential from the dedicated use of land?. Energy Policy..

[48] Berndes G, Abts B, Asikainen A, Cowie A, Dale V, Egnell G, et al. Forest biomass, carbon neutrality and climate change mitigation. From Science to Policy 3. European Forest Institute. 2016.

[49] Höglmeier K, Steubing B, Weber-Blaschke G, Richter K (2015). LCA-based optimization of wood utilization under special consideration of a cascading use of wood. J Environ Manag.

[50] Xu Z, Smyth CE, Lemprière TC, Rampley GJ, Kurz WA (2017). Climate change mitigation strategies in the forest sector: biophysical impacts and economic implications in British Columbia, Canada. Mitig Adapt Strateg Glob Change.

[51] Rüter S. Arbeitsbericht aus dem Institut für Holztechnologies und Holzbiologie 2011/1. Projection of net-emissions from harvested wood products in European Countries.

[52] Pilli R, Grassi G, Kurz WA, Viñas RA, Guerrero NH (2016). Modelling forest carbon stock changes as affected by harvest and natural disturbances. I. Comparison with countries’ estimates for forest management. Carbon Balance Manag.

[53] Pilli R, Fiorese G, Viñas RA, Rossi S, Priwitzer T, Baranzelli C (2016). LULUCF contribution to the 2030 EU climate and energy policy.

[54] Pilli R, Grassi G, Kurz WA, Moris JV, Viñas RA (2016). Modelling forest carbon stock changes as affected by harvest and natural disturbances. II. EU-level analysis. Carbon Balance Manag..

[55] Pilli R, Fiorese G, Grassi G (2015). EU mitigation potential of harvested wood products. Carbon Balance Manag.

